# Topological benchmarking of algorithms to infer gene regulatory networks from single-cell RNA-seq data

**DOI:** 10.1093/bioinformatics/btae267

**Published:** 2024-04-16

**Authors:** Marco Stock, Niclas Popp, Jonathan Fiorentino, Antonio Scialdone

**Affiliations:** Institute of Epigenetics and Stem Cells, Helmholtz Zentrum München—German Research Center for Environmental Health, Munich 81377, Germany; Institute of Functional Epigenetics, Helmholtz Zentrum München—German Research Center for Environmental Health, Munich 85764, Germany; Institute of Computational Biology, Helmholtz Zentrum München—German Research Center for Environmental Health, Munich 85764, Germany; TUM School of Life Sciences Weihenstephan, Technical University of Munich, Munich 85354, Germany; Institute of Epigenetics and Stem Cells, Helmholtz Zentrum München—German Research Center for Environmental Health, Munich 81377, Germany; Institute of Functional Epigenetics, Helmholtz Zentrum München—German Research Center for Environmental Health, Munich 85764, Germany; Institute of Computational Biology, Helmholtz Zentrum München—German Research Center for Environmental Health, Munich 85764, Germany; Institute of Epigenetics and Stem Cells, Helmholtz Zentrum München—German Research Center for Environmental Health, Munich 81377, Germany; Institute of Functional Epigenetics, Helmholtz Zentrum München—German Research Center for Environmental Health, Munich 85764, Germany; Institute of Computational Biology, Helmholtz Zentrum München—German Research Center for Environmental Health, Munich 85764, Germany; Institute of Epigenetics and Stem Cells, Helmholtz Zentrum München—German Research Center for Environmental Health, Munich 81377, Germany; Institute of Functional Epigenetics, Helmholtz Zentrum München—German Research Center for Environmental Health, Munich 85764, Germany; Institute of Computational Biology, Helmholtz Zentrum München—German Research Center for Environmental Health, Munich 85764, Germany

## Abstract

**Motivation:**

In recent years, many algorithms for inferring gene regulatory networks from single-cell transcriptomic data have been published. Several studies have evaluated their accuracy in estimating the presence of an interaction between pairs of genes. However, these benchmarking analyses do not quantify the algorithms’ ability to capture structural properties of networks, which are fundamental, e.g., for studying the robustness of a gene network to external perturbations. Here, we devise a three-step benchmarking pipeline called STREAMLINE that quantifies the ability of algorithms to capture topological properties of networks and identify hubs.

**Results:**

To this aim, we use data simulated from different types of networks as well as experimental data from three different organisms. We apply our benchmarking pipeline to four inference algorithms and provide guidance on which algorithm should be used depending on the global network property of interest.

**Availability and implementation:**

STREAMLINE is available at https://github.com/ScialdoneLab/STREAMLINE. The data generated in this study are available at https://doi.org/10.5281/zenodo.10710444.

## 1 Introduction

Single-cell transcriptomics techniques allow probing patterns of gene expression on an increasingly larger scale, with recent studies including millions of cells and thousands of genes (Svensson *et al.* 2020). Such rapid progress in expanding the scale of available data makes single-cell datasets more appealing for tasks like the inference of gene regulatory networks (GRNs), with the goal of achieving a mechanistic understanding of the systems at hand and going beyond purely descriptive characterizations (Akers and Murali 2021, Stumpf 2021, Saint-Antoine and Singh 2020). However, GRN inference from single-cell data entails many computational challenges, such as high levels of technical noise in the data (Brennecke *et al.* 2013), the extreme sparsity of the ground truth network to be inferred (Banf and Rhee 2017) and the increasing scale of gene expression data (Hillerton *et al.* 2022). For this reason, many algorithms for GRN inference from single-cell data have been published in the last few years. The increasingly large number of such algorithms demands benchmarking studies that can guide the user in the choice of the best-performing methods under various conditions (Chen and Mar 2018, Pratapa *et al.* 2020, Kang *et al.* 2021).

While the benchmarking studies that have been published offer some guidance for users, they are affected by important limitations. First, the quantification of the performance is obtained for a limited number and types of networks. Furthermore, the available benchmarking studies mostly focus on the ability of the GRN algorithms to predict local features of networks, like the interactions between pairs of genes, using, e.g., area under the curve metrics, or the presence of specific sub-graphs (network motifs). These metrics do not assess the algorithms’ ability to infer the structural properties of the GRN, which can quantify important features like the robustness to perturbations (Guo and Amir 2021) and the presence of network hubs representing master regulators. Robustness is one of the main characteristics of GRNs (Noman *et al.* 2015), and for this reason, their topology is also studied to improve the robustness of general network structures in other fields, such as wireless sensor networks (Kamapantula *et al.* 2014, Roy *et al.* 2018).

Moreover, the inclusion of network topology in GRN inference methods has also been shown to improve their performance, e.g., using microarray and bulk RNA-seq data (Villaverde *et al.* 2014, Zhang *et al.* 2015). Recently, an algorithm based on a global network centrality measure and local network motifs has been introduced (Liu *et al.* 2022), showing an improvement in inference performance due to the reduction of network redundancy. This class of inference methods is still lacking for single-cell RNA-seq data.

The structural properties of networks can be quantified by topological measurements (Koutrouli *et al.* 2020), including, for instance, the network efficiency and the assortativity. So far, the performance of GRN inference algorithms on the estimation of topological properties has only been assessed with bulk RNA-seq data (Kiani *et al.* 2016, Escorcia-Rodríguez *et al.* 2023), and by employing a limited number of synthetic networks (Kiani *et al.* 2016), which makes it hard to reach robust conclusions for single-cell data.

In this work, we developed STREAMLINE, a three-step benchmarking framework to score the performance of GRN inference algorithms in estimating structural properties of networks from single-cell RNA-seq (scRNA-seq) datasets. The structural properties we considered quantify the information exchange efficiency, which is related to the network’s robustness to perturbations, and the presence and identification of hubs. We used data simulated from hundreds of networks belonging to four classes with different structural properties (Watts and Strogatz 1998, Ouma *et al.* 2018), as well as from a set of curated (Cur) networks extracted from real GRNs (Pratapa *et al.* 2020). In addition to simulated data, we also used real datasets from yeast, mouse, and human (McCalla *et al.* 2023).

We applied STREAMLINE to four GRN inference algorithms chosen among the top-performing ones in predicting gene-gene interactions (Pratapa *et al.* 2020). Our benchmarking analysis provides guidance in the choice of the algorithm for the prediction of network robustness and the identification of hubs. Moreover, our results point to systematic biases in some algorithms, which could indicate ways of improving them.

To facilitate the use of our benchmarking framework, we made it compatible with an existing pipeline (BEELINE (Pratapa *et al.* 2020)), and we made all the code available in a GitHub repository (https://github.com/ScialdoneLab/STREAMLINE).

## 2 Methods

### 2.1 Ground truth networks

#### 2.1.1 Synthetic networks

We use parameter-controlled networks from four different classes as well as the Cur GRNs that have been used in BEELINE (Pratapa *et al.* 2020). The output of the network samplers is a graph *G* with *n* nodes and *m* edges.

##### *2.1.1.1* Random networks

Random networks were created with the Erdös–Renyi *G*(*n*, *p*) model, which outputs a graph with *n* nodes where each pair is connected with probability *p* (Erdős and Rényi 1959). We set *p* so that the expected number of edges equals *m*.

##### *2.1.1.2* Scale-Free networks

Networks with a degree distribution that follows a power law are classified as Scale-Free (Cho *et al.* 2009). Given the parameter α, the expected degree distribution follows:

(1) $$P(d)∼d^{-\alpha }.$$

For directed networks, the in-degree distribution and the out-degree distribution can feature different parameters *α_in_* and *α_out_*. We applied combinations of different in- and out-degrees. The exact values can be found in Supplementary Table S1.

##### *2.1.1.3* Semi-Scale-Free networks

Following the analysis of the degree distributions in known GRNs (Ouma *et al.* 2018), we sampled Semi-Scale-Free networks which feature an out-degree distribution that follows a power law but a uniform in-degree distribution. Additionally, only 50% of the nodes have outgoing edges.

##### *2.1.1.4* Small-World networks

We used the Watts–Strogatz model to sample networks that feature Small-World topology (Watts and Strogatz 1998). The algorithm starts with *n* nodes with degree *k* in a regular lattice and then rewires edges with probability *p*.

##### *2.1.1.5* Curated networks

Curated networks are four known GRNs that were used in BEELINE to evaluate the statistical performance of the GRN inference algorithms (Pratapa *et al.* 2020). These networks are simple models for mammalian cortical area development (mCAD), ventral spinal cord development (VSC), hematopoietic stem cell differentiation (HSC), and gonadal sex determination (GSD).

##### *2.1.1.6* Network sampling

We use the Julia package LightGraphs.jl https://github.com/JuliaGraphs/Graphs.jl/ to sample the networks explained above. The parameters were chosen such that a large variety of structurally different networks is covered.

##### *2.1.1.7* Simulation of single-cell RNA-sequencing data

We simulate single-cell RNA-sequencing data for the synthetic networks using BoolODE (Pratapa *et al.* 2020), a recently developed method that first converts a Boolean model into a set of ordinary differential equations (ODEs), and then, after adding a noise term, performs stochastic simulations of genes’ expression levels. The method was shown to outperform previously developed algorithms to simulate gene expression from ground truth GRNs like GeneNetWeaver (Schaffter *et al.* 2011). In our simulations, we used the same BoolODE parameters and settings that were extensively tested in Pratapa *et al.* (2020). Specifically, we converted each generated synthetic network into a text file containing a set of Boolean rules, which is given as input to the BoolODE Python script (“path data” parameter). For every parameter set of the synthetic networks, we generated data from 100 cells (“num-cells” parameter) with a simulation time of five steps (“max-steps” parameter) for multiple networks using BoolODE. We used the parameter “sample-cells” to sample one cell per simulation. The number of networks and associated parameters can be found in Supplementary Table S1.

#### 2.1.2 Experimental networks

For the benchmarking of GRN inference on experimental single-cell RNA-sequencing data we selected four datasets from human (Han *et al.* 2018), mouse (Shalek *et al.* 2014, Tran *et al.* 2019), and yeast (Gasch *et al.* 2017) and compared the output networks to different types of silver standard networks that were collected by McCalla *et al.* (2023). The properties of the networks and the number of corresponding silver standards can be found in Supplementary Table S2. The silver standard networks were obtained from public databases and the literature. They were derived from ChIP-chip, ChIP-seq, or gene perturbations followed by bulk sequencing, which yielded multiple networks for each organism (McCalla *et al.* 2023). The ESC silver standard network was obtained from manual curation of GRNs found in the literature, as explained in full detail in McCalla *et al.* (2023).

### 2.2 Inference algorithms

We selected the four top-performing algorithms from BEELINE (Pratapa *et al.* 2020) and examined the results using our three-step benchmarking pipeline. Below, we added a brief description of each algorithm.

GRNBoost2: GRNBoost2 (Moerman *et al.* 2019) infers a GRN independently for each gene, by identifying the most important regulators using a regression model. It is an alternative to GENIE3, which uses a similar inference scheme but does not scale to larger datasets due to its runtime. The output of GRNBoost2 is a directed network.

SINCERITIES: SINCERITIES (Papili Gao *et al.* 2018) is a causality-based method that computes temporal changes in the expression of each gene. The GRN is inferred by solving a specifically formulated ridge regression problem. SINCERITIES outputs a directed network.

PIDC: The PIDC inference scheme (Chan *et al.* 2017) is based on partial information decomposition, which is a multivariate information-theoretic measure for triplets of random variables. Since it is symmetric, the resulting network is undirected.

PPCOR: PPCOR (Kim 2015) calculates the partial and semi-partial correlation coefficients for every possible pair of genes. Edges are ranked according to these values and they are undirected. By using the correlation as a sign, it is possible to obtain activatory and inhibitory interactions. However, we did not use this information in our benchmarking framework.

### 2.3 Evaluation of inferred networks

#### 2.3.1 Processing of GRNs

##### *2.3.1.1* Processing of ground truth networks

In all the ground truth networks, self-loops and duplicate edges are removed. For the experimental datasets, genes in the silver standard networks were subset to the genes that appear in the related gene expression dataset.

In directed networks, the largest weakly connected subgraph of the ground truth was used in the analysis. To perform the analysis on undirected networks, the directed ground truth networks were converted to undirected graphs by ignoring the direction of the edges and then the largest connected subgraph was extracted.

##### *2.3.1.2* Processing of inferred networks

For analysis on undirected networks, the inferred directed networks of GRNBoost2 and SINCERITIES are first converted to undirected networks by ignoring the information about the directionality of the edges.

In both the undirected and directed evaluations, duplicate edges and self-loops in the inferred network are removed. Afterwards, the top *k* edges with the highest absolute predicted weight are used to construct the graph for evaluation. The parameter *k* is chosen to be the same as the number of edges in each associated processed ground truth or silver standard network.

If the resulting graph is not weakly connected in the directed evaluation, the largest weakly connected subgraph is extracted. When evaluating undirected networks, the largest connected subgraph is extracted, if the resulting graph is not connected.

#### 2.3.2 Binary edge detection

To statistically benchmark the edge prediction we followed BEELINE in evaluating the *EPr*, defined as the fraction of true positives among the top *k* edges, ranked according to the weight returned by the inference algorithm, where *k* is the number of interactions in the corresponding ground truth or silver standard network. The *EPr* is better suited to classification accuracy on large datasets where the reference networks do not represent the entire ground truth. For the synthetic networks, we additionally report the *AUPRC* and the *AUROC*, as also commonly done in previous benchmarking (Pratapa *et al.* 2020, Chen and Mar 2018).

#### 2.3.3 Graph properties related to information exchange efficiency

For evaluation of the information exchange efficiency in the graphs, we chose three topological properties. The properties are only computed in the evaluation of undirected networks, thus we assume an undirected graph *G* with a set {*N*} of *n* nodes and *m* edges for the following definitions.

##### *2.3.3.1* Average shortest path length

The average shortest path length $\bar{l_{sp}}(G)$ measures by how many links two random nodes are connected on average:

(2) $$\bar{l_{sp}}(G)=\sum_{v,w\in \{N\}}\frac{d(v,w)}{n\cdot (n-1)},$$

where *d*(*v*, *w*) denotes the distance between two nodes *v* and *w*.

##### *2.3.3.2* Global efficiency

The global efficiency $E_{\mathrm{glob}}(G)$ estimates how efficiently information is exchanged in the network on a global scale. This is related to the concept of the vulnerability of networks to the decrease in networks efficiency in case some of the components malfunction (Latora and Marchiori 2001, Boccaletti *et al.* 2006). It is given by:

(3) $$E_{\mathrm{glob}}(G)=\frac{1}{n\cdot (n-1)}\sum_{\begin{array}{c} v\neq w\\ v,w\in \{N\} \end{array}}\frac{1}{d(v,w)}.$$

Since gene regulation can be interpreted as information exchange between nodes in GRN, *E_glob_* is a meaningful quantity to estimate. A more detailed explanation of the relationship between global efficiency and network vulnerability can be found in Latora and Marchiori (2007).

##### *2.3.3.3* Local efficiency

The local efficiency $E_{\mathrm{loc}}(G)$ describes the resistance of the network to perturbation on a small scale (Latora and Marchiori 2001). Similarly to global efficiency, it is linked to the behavior of the network when some of its constituents fail. A more detailed derivation can be found in Latora and Marchiori (2007). The quantity is defined as:

(4) $$E_{\mathrm{loc}}(G)=\frac{1}{n}\sum_{v\in \{N\}}E_{\mathrm{glob}}(G_{v}),$$

where *G_v_* is the subgraph of *G* that only consists of the direct neighbors of *v*. In practice, perturbations are more likely to be caused by changes in neighboring genes, thus the local efficiency can provide valuable information.

##### *2.3.3.4* Evaluation score for graph properties

Since we wanted to preserve the information on whether certain topological features are over- or underestimated, we employed the *MSE* as an evaluation metric. For a property *P_x_* which is being analyzed on ground truth networks $G_{1},G_{2},\ldots ,G_{k}$ and predicted networks $G_{1,\text{inferred}},G_{2,\text{inferred}},\ldots ,G_{k,\text{inferred}}$, *MSE* is computed by:

(5) $$\mathrm{MSE}(P_{x})=\frac{1}{n}\sum_{i=1}^{k}P_{x}(G_{i,\text{inferred}})-P_{x}(G_{i}).$$

Therefore, a positive value of the MSE refers to an overestimation of the property compared to the ground truth and a negative MSE to an underestimation of the ground truth property.

#### 2.3.4 Topological properties related to the hub analysis

For our hub analysis, we selected different graph and node properties that allow for a meaningful structural characterization of hubs in a network. The graph properties defined below are evaluated with the MSE, as introduced before for the information exchange quantities. The node properties are evaluated with the Jaccard coefficient of the detected hubs, as explained below.

##### *2.3.4.1* Graph properties related to hub topology

###### 2.3.4.1.1 Degree assortativity

The preference for a network’s node to attach to others that have a similar degree is captured by the degree assortativity (Newman 2003). It is quantified by the assortativity coefficient $r_{\deg}(G)$:

(6) $$r_{\deg}(G)=\frac{\sum_{i}e_{ii}-\sum_{i}a_{i}b_{i}}{1-\sum_{i}a_{i}b_{i}},$$

with $a_{i}=\sum_{j}e_{ij},\;b_{j}=\sum_{i}e_{ij}$ and *e_ij_* is the fraction of edges from a node with degree *i* to a node with degree *j* from all edges *m* of the graph. For undirected networks, *i* and *j* are total degrees of nodes, whereas for directed networks we report the Assortativity based on the in-degrees *i* and *j* of the nodes. Networks with a negative assortativity coefficient are called disassortative, and networks with a positive $r_{\deg}(G)$ are called assortative. Disassortative networks have a higher tendency to possess hubs, which is an important feature of GRNs that we examine in Section 2.3.

###### 2.3.4.1.2 Degree centralization

The goal of the degree centralization* H*(*G*) is to provide an estimate of how centralized a graph is around the node $v^{*}$ which has the highest degree in the graph (Freeman 1978). It is defined as:

(7) $$H(G)=\frac{1}{H_{\max}}\cdot \sum_{v\in \{N\}}(\deg(v^{*})-\deg(v)),$$

with

(8) $$H_{\max,\text{undirected}}=(n-1)(n-2),$$

(9) $$H_{\max,\text{directed}}=(n-1)(n-1),$$

for undirected and directed networks, where *deg*(*v*) refers to the total degree of a node *v* for undirected networks and to the in-degree for directed networks, where the in centralization is reported. A highly centralized network is focused around a small number of nodes, which could be identified as biologically important.

###### 2.3.4.1.3 Clustering coefficient

The clustering coefficient measures the extent to which nodes in a graph tend to cluster together. It is quantified by the local clustering coefficient $CC_{\text{loc}}(v)$, which measures the fraction of triangles that exist over all possible triangles in the neighborhood of a node *v*:

(10) $$CC_{\text{loc}}(v)=\frac{2\cdot L_{v}}{k_{v}\cdot (k_{v}-1)},$$

and the global clustering coefficient $CC_{\text{glob}}(G)$ (Watts and Strogatz 1998):

(11) $$CC_{\text{glob}}(G)=\frac{1}{n}\sum_{v\in \{N\}}CC_{\text{loc}}(v),$$

which is the average of the local clustering coefficient over the whole network. *L_v_* represents the number of links between the *k_v_* neighbors of node *v*. For directed graphs, *k_v_* includes both parents of the node with edges going from the parent to the node of interest, and children of the node, with edges going from the node of interest to the child node. For our analysis, we focus on the global clustering coefficient, since it captures clustering on a global scale. A network with a larger global clustering coefficient is more interconnected, which can result in more complex gene regulations.

##### *2.3.4.2* Node properties related to hub identification

###### 2.3.4.2.1 Degree centrality

The degree centrality $C_{D}(v)$ evaluates the degree *d* of a node *v* in a network, scaled by the maximum possible degree:

(12) $$C_{D}(v)=\frac{\deg(v)}{n-1}.$$

For undirected graphs, the total degree centrality is reported, where *deg*(*v*) represents the total degree of node *v*. For directed graphs, the out centrality is reported, where *deg*(*v*) refers to the out-degree of node *v*.

###### 2.3.4.2.2 Betweenness centrality

The betweenness centrality $C_{B}(v)$ describes the extent to which nodes are on the shortest path between other nodes. We use a normalized version of the definition by Freeman (1977):

(13) $$C_{B}(v)=\frac{1}{p_{v}}\sum_{u\neq v\neq w}\frac{\eta_{v}(u,w)}{\eta (u,w)},$$

where η(u,w) is the number of shortest paths from *u* to *w* and $\eta_{v}(u,w)$ is the number of shortest paths from *u* to *w* passing through v. It is normalized by dividing by the number of pairs of vertices not including *v*, which is different for undirected and directed graphs:

(14) $$p_{v,\text{undirected}}=(n-1)(n-2)/2,$$

(15) $$p_{v,\text{directed}}=(n-1)(n-2).$$

###### 2.3.4.2.3 PageRank centrality

The PageRank centrality is the output of the PageRank algorithm which is focused on link analysis. The output is a distribution that models the likelihood of reaching any particular node when randomly moving along edges. Details of the algorithm can be found in Page *et al.* (1999).

###### 2.3.4.2.4 Radiality centrality

The radiality centrality $C_{R}(v)$ (Valente and Foreman 1998) considers the global structure of the networks and indicates how connected an individual is in the entire network structure. It is defined as:

(16) $$C_{R}(v)=\underset{x,y\in \{N\}}{\text{max}}d(x,y)+1-\frac{1}{n-1}\sum_{\begin{array}{c} w\in \{N\}\\ w\neq v \end{array}}d(v,w).$$

###### 2.3.4.2.5 Evaluation score for hub identification

First, we ranked the common nodes between the inferred networks and the associated ground truth network according to the centrality metrics defined above. We then labeled the 10% nodes with the highest values of the metrics as hubs. Then, we analyzed the set similarity between the hubs in the ground truth or silver standard Ω_*true*_ and the inferred networks Ω_*inf*_. To this aim, we computed the Jaccard coefficient *J* (Jaccard 1912) for every network, which is given by:

(17) $$J(\Omega_{\mathrm{true}},\Omega_{\inf})=\frac{\left|\Omega_{\mathrm{true}}\cap \Omega_{\inf}\right|}{\left|\Omega_{\mathrm{true}}\cup \Omega_{\inf}\right|}.$$

To compare the performance between different types of networks, we used as an evaluation score the ratio between the Jaccard coefficient *J* computed on the inferred networks and the expected coefficient *J_rand_* for a random predictor. *J_rand_* can be calculated explicitly from the probability *P* of obtaining a given number of hubs, *x*, among randomly selected nodes Ω_*rand*_ (i.e. $x=|\Omega_{\mathrm{true}}\cap \Omega_{\mathrm{rand}}|$):

(18) $$P(x)=\frac{\left(\begin{array}{c} n_{0}\\ x \end{array}\right)\left(\begin{array}{c} n-n_{0}\\ n_{0}-x \end{array}\right)}{\left(\begin{array}{c} n\\ n_{0} \end{array}\right)},$$

where *n* is the total number of nodes and $n_{0}=|\Omega_{\mathrm{true}}|=|\Omega_{\mathrm{rand}}|$ is the number of nodes selected as hubs from the ground truth network or from the random predictor. Using the above expression of *P*(*x*), the expected value of *J_rand_* obtained with a random predictor can be computed as:

(19) $$J_{\mathrm{rand}}=\sum_{x}J(x)P(x),$$

where the sum runs over all the possible values of $x\in [0,n_{0}]$, and *J*(*x*) is the value of the Jaccard coefficient when the size of the intersection is *x*, namely $J(x)=\frac{x}{2n_{0}-x}$ (as it can be easily obtained from the definition of the Jaccard coefficient, Eq. (17)). With large values of *n* and $n_{0}\ll n$, it can be shown that the following approximation holds true (Chung *et al.* 2019): $J_{\mathrm{rand}}∼\frac{n_{0}/n}{2-n_{0}/n}$.

#### 2.3.5 Aggregated ranking of the algorithms

To provide an overall ranking of the algorithms, we first computed a max-scaled score for each metric, over different network realizations for each network type, and then we aggregated the scores into overall scores for the information exchange efficiency, the hub topology, and the hub identification. We also computed a final overall topology score. Below, we describe in detail how the scores are computed.

For the hub topology and the information exchange efficiency, the average of the MSE of each metric *P_x_* is computed for each network type *t* and algorithm a, where |t| refers to the numbers of networks *G* that are part of *t*:

(20) $$S_{1}(P_{x},a,t)=\frac{1}{\left|t\right|}\sum_{G\in t}{\text{MSE}}_{G}(P_{x},a).$$

Then, for each network type, the absolute values of these averages for all algorithms *A* are max-scaled by dividing the values by the score of the best-performing algorithm on this network type:

(21) $$S_{2}(P_{x},a,t)=\frac{S_{1}(P_{x},a,t)}{\underset{i\in A}{\text{max}}\;S_{1}(P_{x},i,t)}.$$

These scores are then again averaged for each algorithm over the |T| different network types *T*, subtracted from 1 to produce a score that increases with the performance. Finally, the scores are max-scaled again between all algorithms by dividing by the highest score of the algorithms to produce the final scores $F(P_{x},a)$ for each metric:

(22) $$S_{3}(P_{x},a)=1-\frac{1}{\left|T\right|}\sum_{t\in T}S_{2}(P_{x},a,t),$$

(23) $$F(P_{x},a)=\frac{S_{3}(P_{x},a)}{\underset{i\in A}{\text{max}}\;S_{3}(P_{x},i)}.$$

For the hub identification measures *P_y_*, the Jaccard ratios to a random predictor $J/J_{\mathrm{rand}}$ are also averaged for every network type and algorithm. From the average, we subtract 1, to produce a score that sets the performance of a random predictor to 0. Afterwards, the scores are also max-scaled by dividing by the best-performing algorithm per network type, summed over the different network types. The remaining negative values are replaced with 0. These scores are then again max-scaled to produce the final scores $F(P_{x},a)$ for the individual hub identification measures:

(24) $$J_{1}(P_{y},a,t)=\frac{1}{\left|t\right|}\sum_{G\in t}\frac{J(P_{y},a)}{J_{\mathrm{rand}}(P_{y})}-1,$$

(25) $$J_{2}(P_{y},a,t)=\frac{J_{1}(P_{y},a,t)}{\underset{i\in A}{\text{max}}\;J_{1}(P_{y},i,t)}.$$

(26) $$J_{3}(P_{y},a)=\text{max}\left(0,\frac{1}{\left|t\right|}\sum_{t\in T}J_{2}(P_{y},a,t)\right),$$

(27) $$F(P_{y},a)=\frac{J_{3}(P_{y},a)}{\underset{i\in A}{\text{max}}\;J_{3}(P_{y},i)}.$$

With the final scores for each metric, the overall information exchange, overall hub topology, and overall hub identification scores *T*_2_ are calculated by summing up the final scores for the associated metrics *P* for each algorithm a and max-scaling by the best algorithm

(28) $$T_{1}(P,a)=\sum_{p\in P}F(p,a).$$

(29) $$T_{2}(P,a)=\frac{T_{1}(P,a)}{\underset{i\in A}{\text{max}}\;T_{1}(P,i)}.$$

The overall topology score is calculated similarly by calculating *T*_2_ with *P* as all metrics together.

#### 2.3.6 Correlation analysis of the different metrics

To analyze the relationship between the performance in the evaluations of the different metrics, we computed Spearman’s correlation coefficient *ρ* between the performance scores computed as detailed below.

We pooled all results from the synthetic data or the experimental data. Then, for the information exchange and hub topology metrics, we used the negative absolute mean signed error (−|MSE|), to have a score that increases with the performance. For the hub identification metrics, we used as a score the Jaccard coefficient ratio to a random predictor ($J/J_{\mathrm{rand}}$). Finally, we defined as performance scores relative to edge prediction the values of *EPr*, *AUROC*, *AUPRC*. In the heatmaps of Supplementary Fig. S1B and C, we crossed out the correlation values corresponding to a *P* value above .01.

## 3 Results

### 3.1 Overview of STREAMLINE

**Figure 1.**
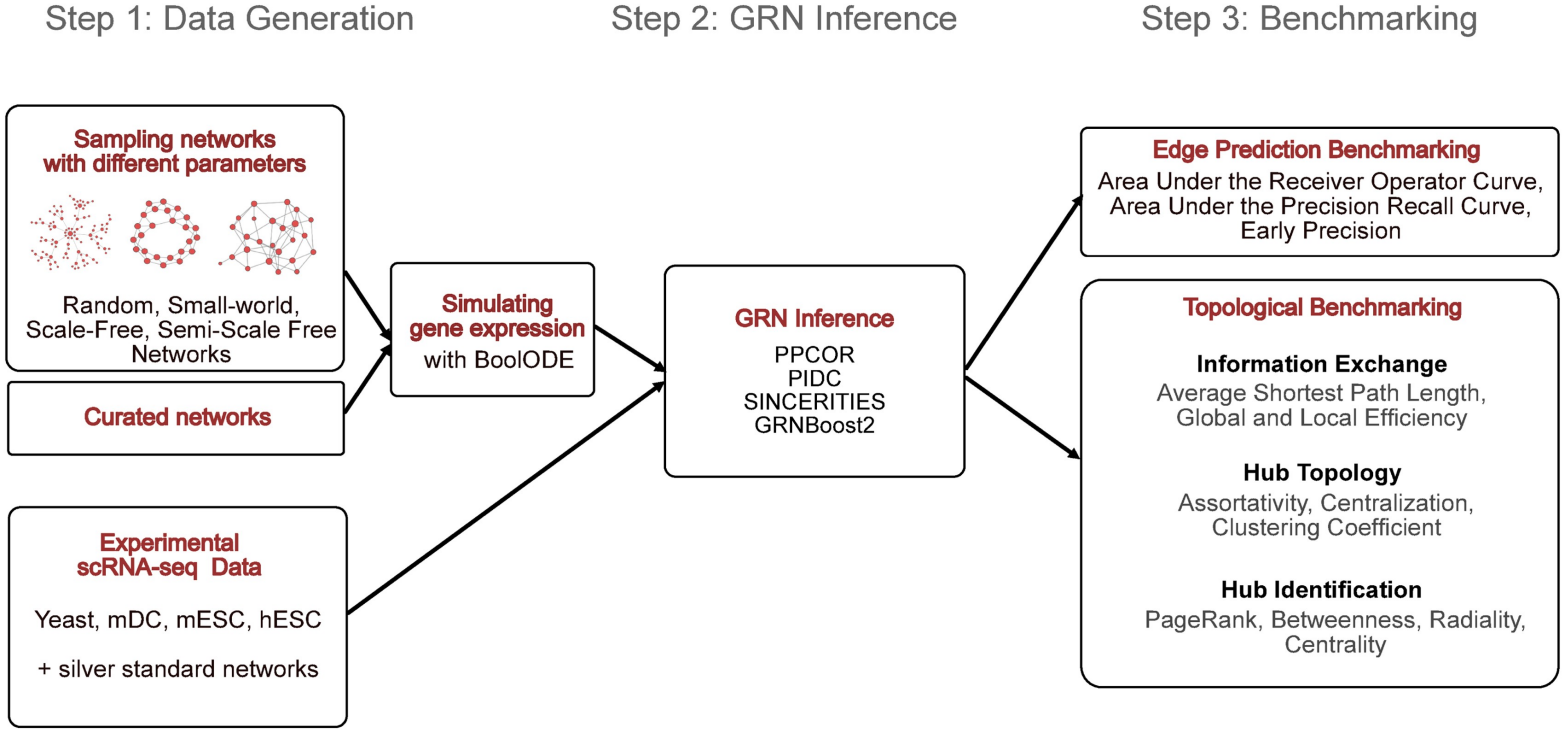
Schematic overview of STREAMLINE. STREAMLINE consists of three steps: first, synthetic scRNA-seq data are generated from different classes of networks (Step 1). Then, GRN inference methods are applied to synthetic as well as real data (Step 2). Finally, the methods’ performance on the predictions of edges and of structural network properties (quantifying the network robustness and hub presence) is evaluated (Step 3).

The steps involved in STREAMLINE are schematically represented in Fig. 1. We consider three types of datasets: simulated, Cur, and experimental datasets.

With the simulated datasets, we generated scRNA-seq data *in silico* from four classes of networks with well-defined and different structural properties, to be able to test the algorithms in different scenarios. The classes of networks we consider are Random, Small-World (SW), Scale-Free (SF), and Semi-Scale-Free (SSF) Networks. Random or Erdös–Renyi (ER) networks include a set of nodes in which each node pair has the same probability of being connected by an edge (Erdős and Rényi 1959). We include this class of networks as a control. In SF networks, the edges are drawn such that the degree distribution follows a power law (Barabási *et al.* 2000). SF networks have been considered ubiquitous in cell biology (Albert 2005), but their presence, at least on a global network level, is still debated (Broido and Clauset 2019). For this reason, we also employ SSF networks, in which only the out-degree distribution follows a power law, while the in-degree distribution is uniform. Such networks were introduced by Ouma *et al.* (2018) to model real GRNs. SW networks have the property that the neighbors of any given node are likely to be neighbors of each other (Watts and Strogatz 1998). The SW property has been observed, for instance, in yeast (Van Noort *et al.* 2004) and human lung cancer (Sun *et al.* 2006) GRNs. In addition to these, we included four Cur networks that consist of sub-networks of known GRNs (Pratapa *et al.* 2020).

Networks from each class are defined by a set of parameters. To make our results independent of specific instances of networks, we sampled multiple networks from each class with different combinations of parameters and two sizes: a smaller (15 nodes and 50 edges) and a larger (25 nodes and 100 edges) size. All the results shown below are averaged over all the instances of networks generated for a given class. Details about the network classes and the parameters used for network sampling are provided in the Methods section, Supplementary Section S1 and Supplementary Table S1. From each of these networks, we simulated scRNA-seq datasets using BoolODE, a recently developed software based on ordinary differential equations (Pratapa *et al.* 2020) (see Methods section).

In addition to simulated datasets, we also considered four real scRNA-seq datasets generated from different organisms and cell types: yeast (Gasch *et al.* 2017), mouse dendritic cells (mDC) (Shalek *et al.* 2014), mouse embryonic stem cells (mESC) (Tran *et al.* 2019), and human embryonic stem cells (hESC) (Han *et al.* 2018). These datasets were used in a previous benchmarking study (McCalla *et al.* 2023), where the authors also provide estimations of silver standard networks. We report the details about the experimental datasets in Supplementary Table S2.

The second step of our pipeline involves running the algorithms to infer GRNs from each of the datasets. We chose the four top-performing algorithms according to a recent study where the accuracy in predicting gene–gene interactions was evaluated (Pratapa *et al.* 2020): PIDC (Chan *et al.* 2017), PPCOR (Kim 2015), SINCERITIES (Papili Gao *et al.* 2018), and GRNBoost2 (Moerman *et al.* 2019). Two of these methods (PIDC and PPCOR) give output as undirected networks, while SINCERITIES and GRNBoost2 provide directed networks. A brief description of each algorithm is included in the Methods section. To make the results comparable between the different algorithms, we considered the undirected version of the networks inferred by SINCERITIES and GRNBoost2. We show the effect of taking the edge direction into account in the Supplementary Material.

In our analysis, we first scored each method’s ability to predict the presence of edges. Specifically, we calculated the early precision (EPr) on the experimental and synthetic data. For the synthetic data, we computed also the area under the receiver operator curve (AUROC) and the area under the precision-recall curve (AUPRC) (Pratapa *et al.* 2020). The results are then grouped for each network class or organism, and we found results in line with previous studies (see (Pratapa *et al.* 2020, Chen and Mar 2018), Supplementary Section S2 and Supplementary Fig. S1).

Then, we analyzed the ability of each method to predict global properties of networks, which are defined at a graph level. In particular, we computed topological properties that quantify how efficiently the information is exchanged in the network and the tendency of networks to include hubs.

The efficiency of information exchange measures how the behavior of a network can change following variations in its topology due to, e.g., the failure of some of its constituents (Latora and Marchiori 2001). In this context, it can be used to assess the stability of a GRN, as it is subject to random errors due to mutations and extreme conditions that can hinder regulatory interactions (Boccaletti *et al.* 2006). The following topological measures can quantify the efficiency of information exchange: the Global Efficiency, the Local Efficiency, and the Average Shortest Path Length (see Methods section). The Global and Local Efficiency measures quantify the fault tolerance of a system (Latora and Marchiori 2007) and they have already been used to study the relationship between evolutionary and topological properties of human GRNs (Szedlak *et al.* 2016). The Average Shortest Path Length has been widely adopted as a measure of biological network navigability (defined as the ability to efficiently move from a source to a target node through short communication paths), which is crucial for information distribution (Barabasi and Oltvai 2004, Boguñá *et al.* 2009). Another biologically important property of networks is the presence of hubs, i.e., nodes that have a degree much larger than the average. In GRNs, hubs are genes that regulate the expression levels of many other genes and can represent master regulators of a biological process. Through structural network analysis, it has been shown that the presence of hubs is highly sensitive to perturbations in network topology (Ghoshal and Barabasi 2011), and it is linked to global topological quantities like the Centralization, the Assortativity, and the Clustering Coefficient (Sporns *et al.* 2007, Pechenick *et al.* 2012), which we compute in STREAMLINE.

In addition to quantifying the tendency of networks to possess hubs, it is important to identify them correctly. Hence, we tested the GRN inference algorithms for their ability to predict which nodes constitute hubs. To do so, we computed four local metrics used to detect hubs (Koschützki and Schreiber 2008)—Page Rank Centrality, Betweenness Centrality, Centrality, and Radiality (see Methods section)—and we compared the values obtained from the ground truth networks versus those calculated from the inferred networks.

Below, we describe the detailed results of each of these benchmarking analyses.

### 3.2 Estimation of information exchange efficiency

**Figure 2.**
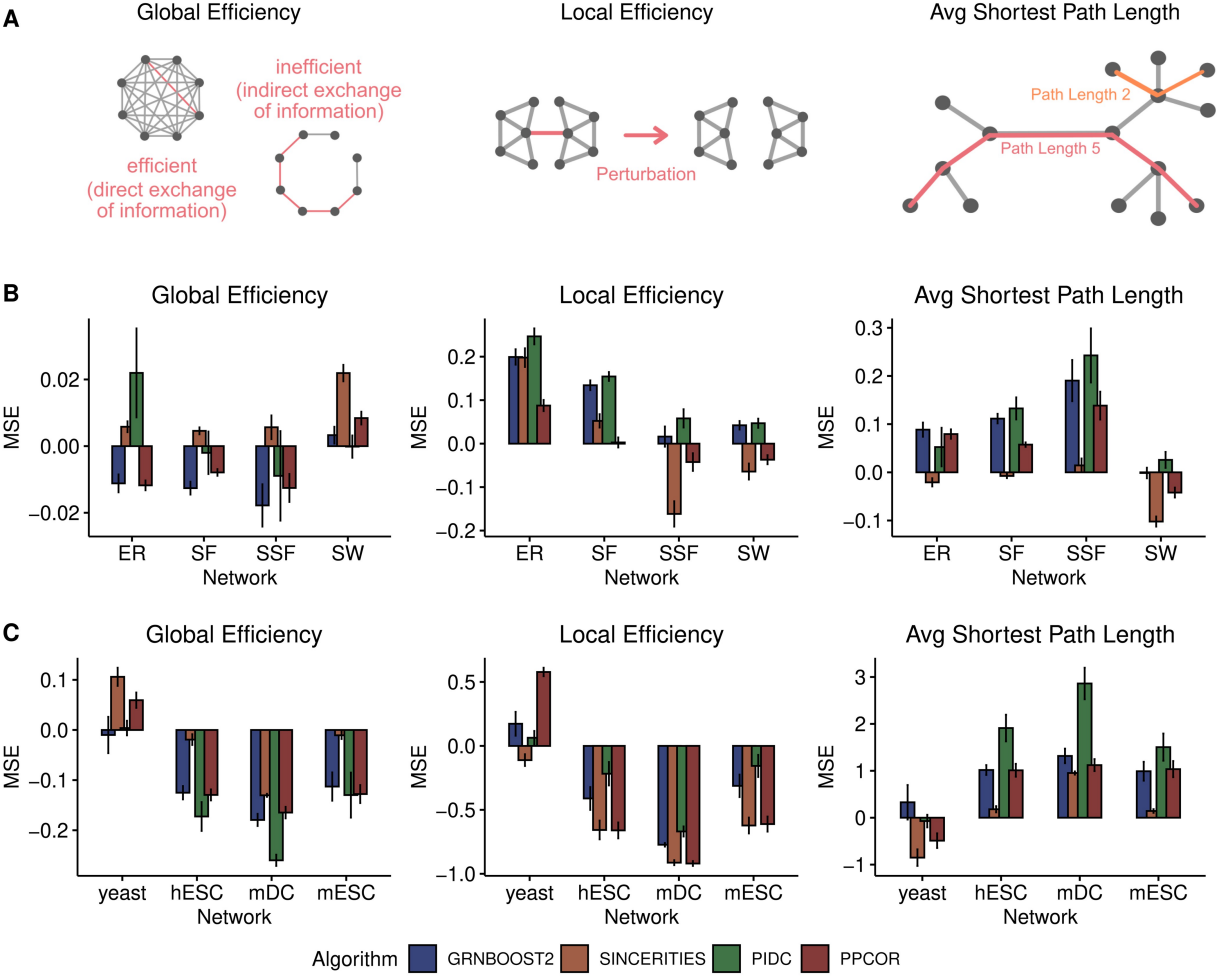
Results of the topological benchmarking of GRN inference algorithms with respect to information exchange both on synthetic and experimental scRNA-seq datasets. (A) Schematic representations of the three topological measures we computed (see Methods section). Global Efficiency quantifies how well the information can be distributed in the entire network. Local Efficiency measures how robust the network is to perturbation on a small scale. The Average Shortest Path Length specifies how many links are necessary to go from one node to another on average. (B) Barplots showing the mean signed error (MSE) for the estimations of the topological properties written at the top in different types of synthetic networks (indicated on the *x*-axis) and for different algorithms (marked by colors). (C) Same as B, for networks estimated from real scRNA-seq datasets (indicated on the *x*-axis). The heights and the error bars display the mean of the MSE values and the standard error of the mean, respectively, computed across datasets and networks in panel B and across networks in panel C.

To quantify the efficiency of information exchange, we evaluated the Average Shortest Path Length, the Global Efficiency, and the Local Efficiency (Fig. 2A) of the inferred and ground truth networks. Then, we quantified the accuracy of the estimations obtained from the inferred networks by calculating the mean signed error (MSE) between these quantities computed on the ground truth networks and the networks inferred from each of the algorithms (see Methods section).

First, we considered the simulated datasets generated from different classes of networks. The different structural properties of each class of networks are reflected by different values of these topological measures, as shown in Supplementary Fig. S2A. For example, the SW networks are characterized by a larger Clustering Coefficient and higher Global and Local Efficiency compared to ER networks, as expected based on their properties (Watts and Strogatz 1998).

In Fig. 2B, we report the MSE for all the topological measures computed on the simulated datasets. With Global Efficiency, we observed relatively accurate estimations for all the synthetic networks with all the algorithms (the absolute value of the average MSE per network type is ∼3% of the ground truth values; see Supplementary Fig. S2A). However, we observed that GRNBoost2 and PPCOR tend to underestimate it in ER, SF, and SSF networks, while SINCERITIES always overestimates it. The dependence on the type of network is particularly evident for some algorithms, like PIDC: while it provides the most accurate estimation of Global Efficiency in SW networks (MSE ∼0), it shows the worst performance with ER networks (where the associated MSE is the largest).

We saw similar variability in the estimations of Local Efficiency. All the tested algorithms tend to overestimate it (MSE > 0), except for SINCERITIES and PPCOR, which underestimate it in SSF, SW, and Cur networks (Supplementary Fig. S3 and Supplementary Table S3). The best predictions (corresponding to MSE∼0) are obtained on SW and SSF networks.

For the Average Shortest Path Length (Fig. 2B) SINCERITIES and PPCOR provide the best estimations, especially in the ER, SF, and SSF networks, while PIDC and GRNBoost2 perform better for SW networks. In the Cur networks, for which the Average Shortest Path Length is greater than for SSF graphs (Supplementary Fig. S2A and Supplementary Table S3), the algorithms tend to underestimate this property.

We performed the same analysis on four real scRNA-seq datasets from three species (Fig. 2C). The corresponding silver standard networks have lower Global Efficiency, similar Local Efficiency, and larger Average Shortest Path Length compared to the synthetic networks we considered, except for the yeast dataset that stands out for its lower values of the Local and Global Efficiency (Supplementary Fig. S2B).

The corresponding values of MSE are reported in Fig. 2C. Interestingly, we found an overall tendency of all algorithms to underestimate both the Local and Global Efficiency, except for the yeast dataset. SINCERITIES provides the most accurate predictions of the Global Efficiency in the hESC, mDC, and mESC networks, while PIDC outperforms the other algorithms for the yeast dataset. PIDC is also the top-performing algorithm for Local Efficiency (Fig. 2C).

As for the Average Shortest Path Length, the MSE is mostly positive, indicating an overestimation, and it is smallest for SINCERITIES, which is the best-performing algorithm for all networks except for the yeast dataset, where it is outperformed by PPCOR.

Overall, the analysis above shows that the accuracy of the estimations of the topological properties measuring the information exchange depends both on the type of network and the algorithm. Moreover, at least with synthetic networks, properties like Global Efficiency are estimated with a small relative error (∼3%), while the relative errors on the other properties and with experimental networks can be much larger (Fig. 2B and C and Supplementary Fig. S2A).

Next, we checked whether the performance of the algorithms in predicting these topological properties correlated with their ability to predict edges in the network. Interestingly, we found that the correlation between the performance measured in these two tasks is either statistically non-significant or very small (Supplementary Fig. S1B and C).

### 3.3 Hub analysis

One important downstream analysis on GRNs is the identification of genes with a number of links much larger than the average. These are known as network hubs, which can play key roles in differentiation and reprogramming (Kim *et al.* 2021) and have been identified as potential disease regulators or drug targets (Åkesson *et al.* 2021). The presence of hubs depends on several topological properties that change with the type of network. For example, we expect the hubs in SF and SSF networks to be more easily identifiable due to their node-degree distribution. Such a feature of SF and SSF networks is reflected by their higher Centralization values compared to other classes of networks (Supplementary Fig. S2A).

Here, we analyzed how accurately the algorithms can predict the values of two groups of topological properties: the first group of graph properties quantifies the tendency of networks to include hubs (Assortativity, Clustering Coefficient, and Centralization), and the second group of node properties is used to identify hubs (Betweenness, Centrality, Radiality, and PageRank). For hub identification, we use random networks for baseline predictions.

#### 3.3.1 Hub-related topological quantities

**Figure 3.**
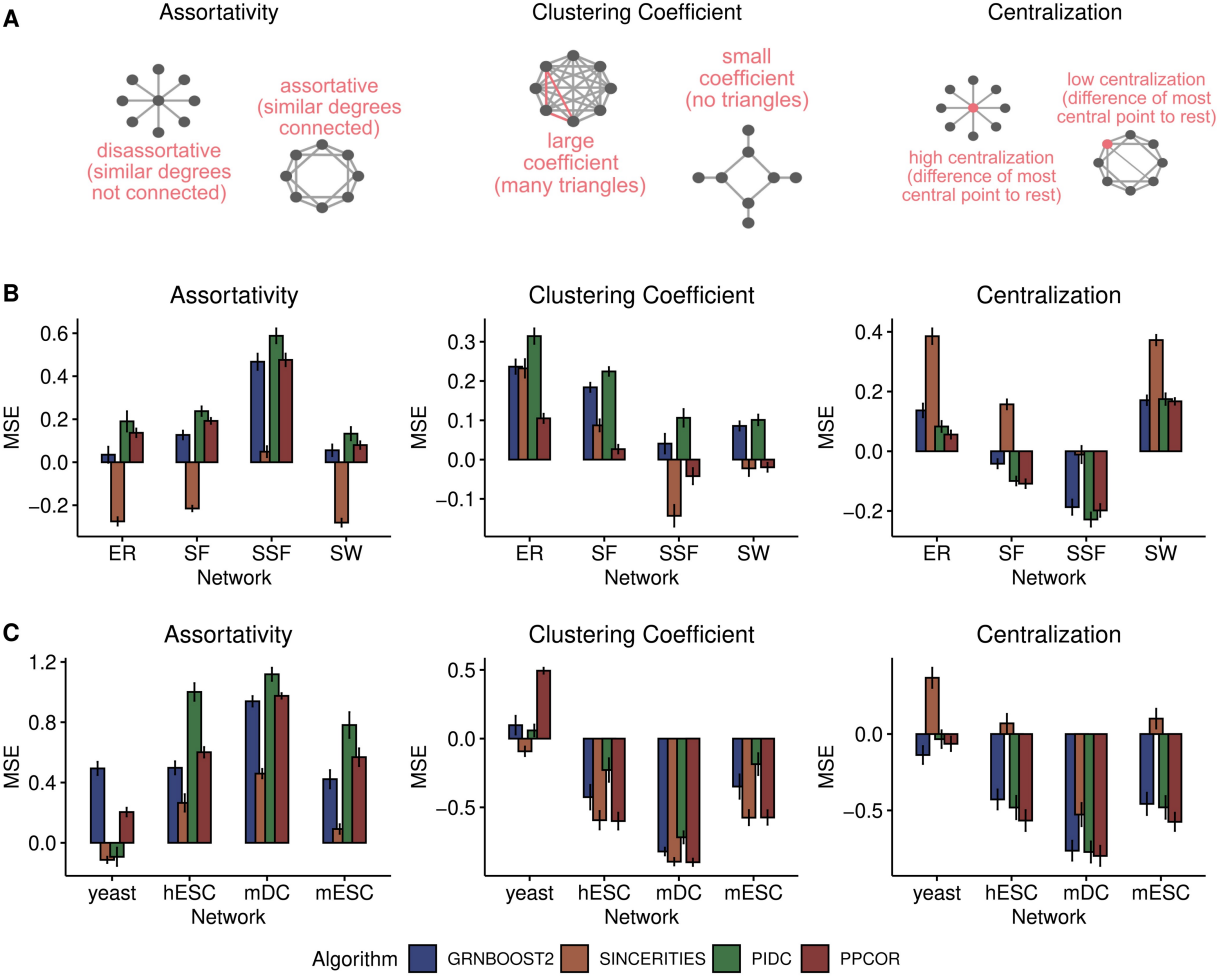
Results for the topological benchmarking of GRN inference assessing the presence of hubs. (A) Schematic representation of the three topological measures considered here (see Methods section). The Assortativity quantifies the tendency of nodes in the networks to attach to others with similar degrees. The Clustering Coefficient reflects how much the nodes in a graph tend to cluster together. The Centralization indicates how strongly the network is arranged around a single center. (B) Barplots showing the mean signed error (MSE) for the estimations of the topological properties written at the top in different types of synthetic networks (indicated on the *x*-axis) and for different algorithms (marked by colors). (C) Same as B, for networks estimated from real scRNA-seq datasets (indicated on the *x*-axis). The heights and the error bars display the mean of the MSE values and the standard error of the mean, respectively, computed across datasets and networks in panel B and across networks in panel C.

The topological measures we chose to quantify the presence of hubs are Assortativity, Clustering Coefficient, and Centralization (Fig. 3A). In networks with negative and larger absolute values of Assortativity, nodes with lower degrees tend to be linked to nodes that feature a higher degree; hence, in these networks, hubs tend to be present and clearly identifiable. Networks with a large Clustering Coefficient feature groups of nodes with high interconnectivity that, thus, have similar node degrees. In this situation, hubs are less dispersed. The Centralization quantifies how centralized a graph is around a small number of nodes, which will have a large number of links, and will therefore tend to be strong and clearly identifiable hubs.

The synthetic networks we simulated data from are characterized by different values of the above topological measures due to their properties (see (Ouma *et al.* 2018), Supplementary Fig. S2A for undirected networks and Supplementary Fig. S4A for directed networks). This allowed us to explore the performance of the algorithms under different conditions. The three network properties assessed during this step uncovered multiple algorithm-specific behaviors (Fig. 3B). The most evident involves the algorithm SINCERITIES, which yielded GRNs with lower Assortativity (Supplementary Fig. S2A), which leads to an underestimation of this property for almost all types of networks, including the Cur networks (Fig. 3B and Supplementary Table S3). The results for the Clustering Coefficient are similar to those obtained for the Local Efficiency, with an overestimation of this property in ER and SF networks, and better performance of the algorithms in the case of SSF and SW networks (Fig. 3B). This result is in line with a known general relationship between these two metrics (Strang *et al.* 2018). In the case of the Cur networks, we also observed a tendency to overestimate the Clustering Coefficient, although the behavior is more dataset-specific (Supplementary Table S3).

When using directed networks, we found that the estimations of the Clustering Coefficient by GRNBoost2 and SINCERITIES change only slightly, while for the Assortativity we observed marked differences for SINCERITIES, which overestimates this property in all networks (Supplementary Fig. S4C).

As in the previous section, we repeated the analysis using real datasets (Fig. 3C). The corresponding silver standard networks have Assortativity values that are lower than those of most synthetic networks and are in line with the SF hypothesis for GRNs (Ouma *et al.* 2018). On average, the Clustering Coefficients are similar to those of SF and SSF networks (Supplementary Fig. S2B), except for the yeast dataset that shows smaller values of this metric. Such values of the topological properties indicate that these networks display a higher tendency to contain hubs that are more clustered together when compared to random networks.

Similarly to what happens with the synthetic datasets, here, the Centralization is overestimated by SINCERITIES (Fig. 3C), except for the mDC networks that are more centralized (Supplementary Fig. S2B). In contrast to the synthetic networks, the Assortativity is now overestimated by SINCERITIES rather than being underestimated.

GRNBoost2, PIDC, and PPCOR show similar performances. These three algorithms overestimate the Assortativity and underestimate the Centralization. Furthermore, the Clustering Coefficient is underestimated by all algorithms for the hESC, mDC, and mESC datasets, while the estimations are more accurate for the yeast dataset, whose silver standard networks have smaller values of this metric (Supplementary Fig. S2B).

Overall, we found differences in performance between the real and the synthetic data, which might be due to a number of factors. First, the silver standards provide only estimates for GRNs of the three organisms, while the synthetic data are simulated from fully specified networks. Furthermore, we found that the algorithms tend to output networks that feature similar values for the topological properties, regardless of the type of network they are run on (Supplementary Fig. S2). This might explain, e.g., the opposite trends in the estimations of the Assortativity and the Clustering Coefficient with the synthetic versus the real datasets, as observed in particular for the SINCERITIES algorithm.

Finally, we checked whether the algorithms’ ability to estimate the hub-related topological quantities correlates with their performance in predicting network edges. Consistently with what we observed before when looking at information exchange (see previous section), we found little to no correlation (Supplementary Fig. S1B and C).

#### 3.3.2 Hub identification

While hubs are loosely defined as nodes having degrees higher than average, there is no consensus on the best metric to identify them. For this reason, here we compute four centrality measures that have been previously adopted to find hubs in GRNs (Koschützki and Schreiber 2008): the Betweenness (Freeman 1977), the Centrality (Freeman 1978), the Radiality (Valente and Foreman 1998), and the Page Rank (Page *et al.* 1999) (see Methods section). Among these, the Page Rank and the Centrality metrics are conserved along evolution and relevant in pluripotent cells (Wolf *et al.* 2021). Moreover, they were proposed as metrics to distinguish life-essential versus specialized subsystems (Wolf *et al.* 2021).

**Figure 4.**
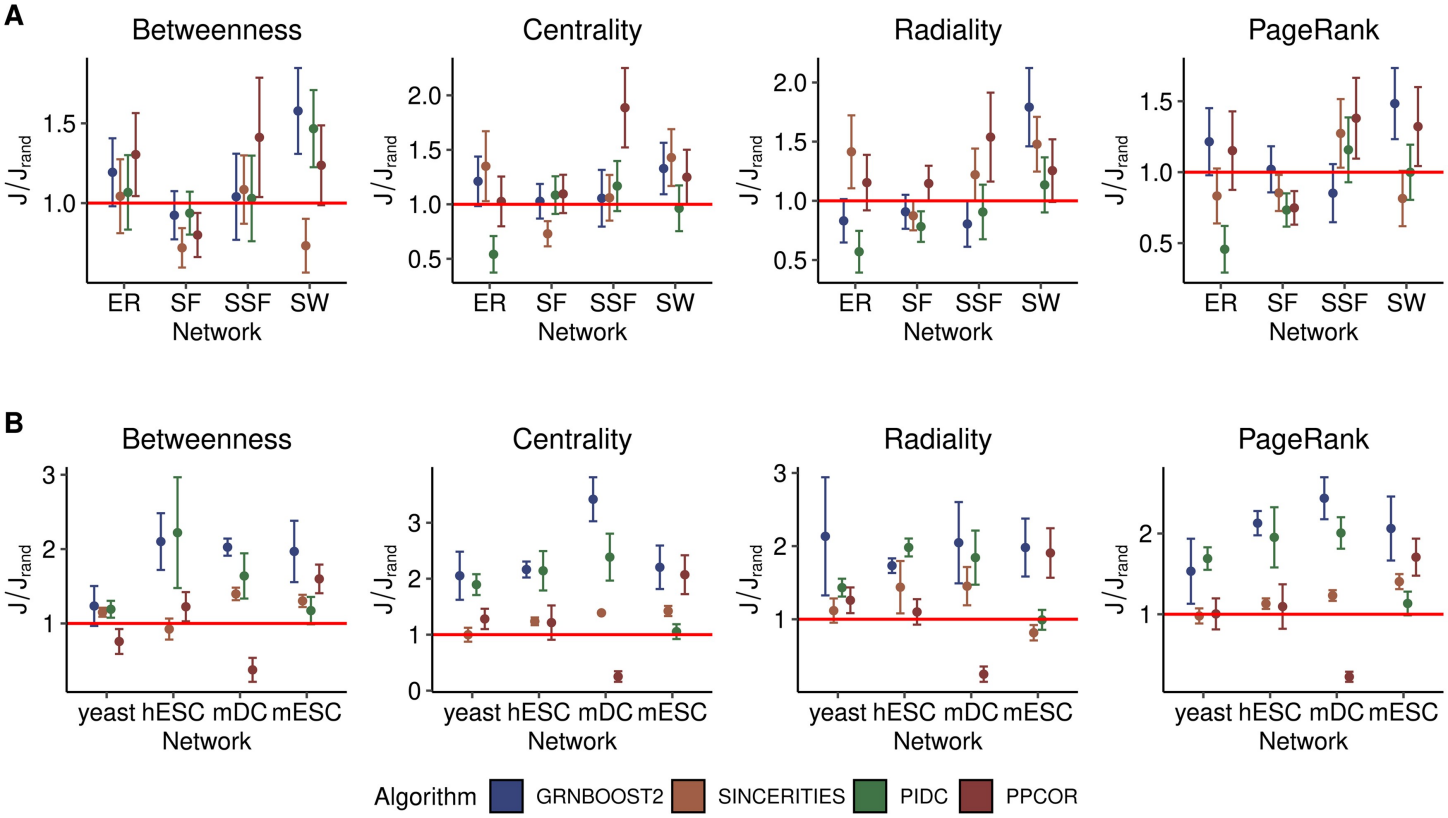
Accuracy of hub detection. The accuracy is measured by the Jaccard coefficient ratio, $J/J_{\mathrm{rand}}$, using a random predictor as a reference (see Methods section). (A) $J/J_{\mathrm{rand}}$ is plotted for various hub metrics (written on top) as a function of the type of synthetic network (indicated on the *x*-axis) for different algorithms. (B) Same as (A), plotted as a function of the networks inferred from real scRNA-seq datasets (indicated on the *x*-axis). The Betweenness estimates the influence that a node has on the information exchange in a graph based on path lengths. The Centrality is the normalized total degree of a node. The Radiality assigns high centrality values to nodes with a short distance to all vertices in their reachable neighborhood compared to the graph diameter. PageRank is a generalization of the degree centrality that considers the eigenvalues of a modified adjacency matrix. We provide a detailed definition of the hub metrics in the text and the Methods section. The points and the error bars display the mean of the Jaccard coefficient ratio and the standard error of the mean, respectively, computed across datasets and networks in panel A and across networks in panel B.

We verified how accurately the hub identification measures are estimated by the four inference algorithms introduced above. More specifically, we selected the set of top 10% nodes according to the centrality measure computed in the ground truth network, Ω_*true*_, and in the inferred network, Ω_*inf*_. Then, we quantified the similarity between the two sets of nodes with the Jaccard coefficient, *J* (Jaccard 1912) (see Methods section). Finally, we computed the ratio between *J* and *J_rand_*, i.e., the Jaccard index between Ω_*true*_ and a set of randomly selected nodes Ω_*rand*_ (see Methods section). Hence, the ratio $J/J_{\mathrm{rand}}$ shown in Fig. 4 represents how well the hubs can be predicted from the inferred networks with respect to a random guess for synthetic (panel A) and experimental (panel B) networks.

In synthetic networks, we obtain similar values of the Jaccard coefficient on average (Supplementary Fig. S5A) but lower values of $J/J_{\mathrm{rand}}$ (Fig. 4A), compared to the experimental networks (Fig. 4B). Better performances are obtained in SSF networks, which are the most centralized, and SW networks, while the performances are generally poor for the SF networks. These results are likely related to the underestimation for SF networks and overestimation for the SW networks of the values of the Centralization that we previously observed (Fig. 3B and Supplementary Fig. S2A).

For the experimental networks, we find that higher values of $J/J_{\mathrm{rand}}$ are achieved by GRNBoost2 and PIDC in networks with stronger hubs (mDC, hESC, and mESC) (Fig. 4B), which features larger values of the Centralization and Clustering Coefficient compared to the yeast network (Supplementary Fig. S2B). However, the values of the Jaccard coefficients, *J*, are smaller than 0.2, indicating an overall poor performance of the algorithms (Supplementary Fig. S5B). PIDC and GRNBoost2 emerge as the top-performing algorithms, especially in the hESC and mDC networks, depending on the hub identification metric.

When we ran the analysis on directed networks, we found similar performance on synthetic networks, while for the experimental networks we observed a large increase of $J/J_{\mathrm{rand}}$, especially for GRNBoost2 when using the Betweenness or the Out Centrality (Supplementary Fig. S6). This result is in line with recent results on hub identification from experimental scRNA-seq data (Kim *et al.* 2021, Fiorentino *et al.* 2024).

## 4 Discussion

Here, we performed benchmarking analyses to evaluate how well GRN inference algorithms can estimate the structural properties of the networks. More specifically, we quantify the ability of the algorithms to infer their robustness to perturbations as well as the presence and identification of network hubs. For this purpose, we computed six topological measures and tested four metrics for hub identification that are widely known and used in network theory. Moreover, we considered scRNA-seq data simulated from different types of networks as well as real data collected from different organisms.

In this extensive benchmarking, we focused on network properties (i.e. robustness and hubs) that are not taken into account in currently available benchmarking studies performed on scRNA-seq data, despite being considered important when studying GRNs and, more in general, biological networks (see, e.g. Noman *et al.* 2015, MacNeil and Walhout 2011, Winterbach *et al.* 2011). For example, the identification of putative master regulators via degree-based measures on GRNs is a commonly used practice (see, e.g. Koschützki and Schreiber 2008, Cholley *et al.* 2018, Padi and Quackenbush 2015). We chose to focus on general topological properties of networks whose definition and interpretation do not require assumptions on the biological process under study. However, more targeted approaches to investigate, e.g., network robustness, such as the *in silico* perturbation of specific genes (Theodoris *et al.* 2023) might be included in STREAMLINE in the future.

**Figure 5.**
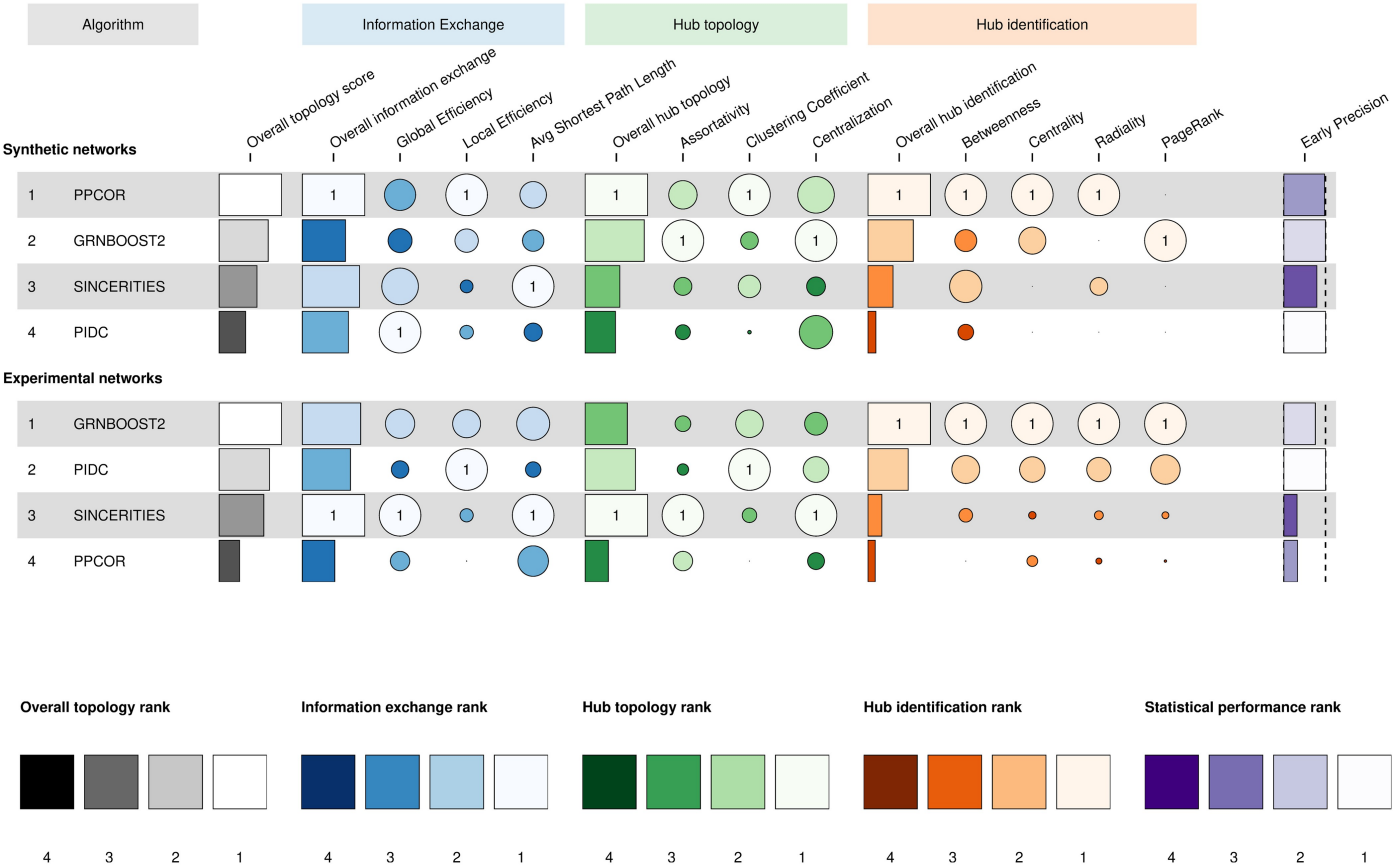
Ranking of the GRN inference algorithms. We report the overall performance of the algorithms on each topological metric for synthetic (top rows) and experimental (bottom rows) datasets. The algorithms are ranked according to an overall topology score (see Methods section). We also show the ranking for each group of topological metrics (Information Exchange, Hub Topology, and Hub Identification) and we report the performance in binary edge detection in the last column. The legend at the bottom shows the association between the colors and the ranks for each group of metrics.

The benchmarking results are summarized in Fig. 5 (for undirected networks) and Supplementary Fig. S7 (for directed networks).

Interestingly, we found that the algorithms’ performance in edge detection (Fig. 5 and Supplementary Fig. S1) has weak or no correlation with their performance in estimating the topological properties of networks, which indicates the need for a targeted benchmarking analysis like STREAMLINE. Moreover, this result also implies that a GRN inference algorithm with poor performance in edge prediction can still provide accurate estimates of global network properties.

For synthetic datasets, we found that PPCOR is the best-performing algorithm in the three tasks that we benchmarked for topological metrics: Information Exchange, Hub Topology, and Hub Identification. However, we highlight that other algorithms might be preferred for the estimation of specific topological metrics. Indeed, SINCERITIES emerges as the top-performing algorithm for estimating the Average Shortest Path Length and GRNBoost2 has the best performance in estimating the Assortativity and the Centralization in the Hub Topology task, and the PageRank metric in the Hub Identification task.

In the case of experimental networks, we found that the best-performing algorithm is more dependent on the specific topological metrics (Fig. 5). While GRNBoost2 provides the best estimates for all the metrics related to Hub Identification, SINCERITIES is the top-performing one for the metrics related to the Information Exchange and Hub Topology. The Local Efficiency and Clustering Coefficient, for which we observed that the estimates are closely related for all algorithms (Figs. 2 and 3), are an exception since they are estimated better by PIDC.

We also found that, with directed networks, GRNBoost2 overall performs better than SINCERITIES (Supplementary Fig. S7) and the identification of hub genes is generally more accurate, as suggested by the higher values of $J/J_{\mathrm{rand}}$ (Fig. 4 and Supplementary Fig. S6).

The benchmarking done with synthetic networks allowed us to check the performance of algorithms with networks having specific and tunable properties. In some cases, this has brought to light specific biases present in the networks estimated by each algorithm. In particular, for most of the algorithms the inferred average values of some metrics (e.g. Assortativity and Local Efficiency) for different types of synthetic networks are close and do not show any clear trend, unlike their ground truth values (Supplementary Fig. S2). In other cases, the trend can even be inverted, as in the case of the Clustering Coefficients estimated by SINCERITIES, which are highest on average for ER networks and decrease in SF and SSF networks, while the ground truth values show the opposite trend (Supplementary Fig. S2).

Some algorithms show specific features. For example, SINCERITIES produces more disassortative and centralized networks (i.e. networks with relatively low Assortativity and high Centralization), which causes an underestimation of Assortativity and overestimation of Centralization for all types of synthetic networks (Fig. 3A). Similar observations can be made, e.g., with GRNBoost2, which tends to generate networks with lower Global Efficiency (Fig. 2A). While the underlying reasons for these observations remain elusive, we speculate that they might be caused by differences in the specific designs of the algorithms. For instance, SINCERITIES is a causality-based method that uses a linear regression model on temporal data, similar to Granger causality, which is known to have high false positive rates when its underlying assumptions are violated, as is the case in complex datasets with nonlinear dynamics (Yuan and Shou 2022). More specifically, Granger causality fails when the system’s dynamics are deterministic (or have a low noise level) or when pairs of variables have a common unobserved cause (Yuan and Shou 2022). The reason why SINCERITIES tends to output disassortative and centralized networks might come from the preferential attribution of false positive edges to specific genes, due to their specific dynamics.

Importantly, the knowledge of such biases can guide the effort to improve current algorithms, e.g., by assisting in the design of objective functions that can lead to networks with global properties closer to real GRNs. This approach can be justified by the observation that GRNs share certain topological features, such as an SF (Lopes *et al.* 2014) or SSF (Ouma *et al.* 2018) node-degree distributions, which could be assumed as prior knowledge during the inference process.

In this study, we chose to focus on unsupervised GRN inference algorithms, which are currently widely used by the community and are well characterized in their performance with local metrics (Pratapa *et al.* 2020, Chen and Mar 2018). It will be interesting to include in future versions of STREAMLINE the benchmarking of (semi-)supervised methods for GRN inference and the calculation of global metrics taking into account edge weights.

The precise definition of the ground truth has crucial importance in benchmarking studies. Current studies rely on either simulated data or experimental silver standard networks. While these methods represent the state-of-the-art, it is essential to acknowledge the limitations associated with both types of ground truths. Simulated data faces constraints related to the parametrization of ground truth networks into ODE or SDE models, as exemplified by BoolODE, which could affect network identifiability (Erbe *et al.* 2023). Nonetheless, innovative approaches are emerging, leveraging mechanistic models of gene regulation (Erbe *et al.* 2023), or deep-learning-based models (Zinati *et al.* 2023). These advancements aim to directly generate scRNA-seq datasets that encode direct causal regulatory relationships. Conversely, experimental silver standard networks are typically derived from ChIP-seq experiments. These experiments, while valuable, come with known limitations, including sequencing errors and GC bias (Park 2009). Such limitations may result in missing or inaccurate edges within the ground truth network. Furthermore, the incompleteness of these networks can affect the estimation of topological properties. These limitations are shared across benchmarking studies. However, the development of robust, widely applicable computational pipelines, such as STREAMLINE, is essential for ongoing enhancements in ground truth network generation and paves the way for more accurate assessments in the evolving landscape of benchmarking studies.

Finally, the topological quantities we considered can also be used to optimize community-based inference schemes. Currently, consensus networks are derived from the outputs of different methods by taking into account only their performance in estimating edges. Instead, new strategies could be devised that also consider the estimation of the network’s topological properties.

## Supplementary Material

btae267_Supplementary_Data

## Data Availability

We used publicly available experimental single-cell RNA-sequencing data from yeast (Gasch *et al.* 2017), mouse (Shalek *et al.* 2014, Tran *et al.* 2019), and human (Han *et al.* 2018) with Cur silver standard networks from McCalla *et al.* (2023). The normalized gene expression, the pseudo time data and silver standard networks can be downloaded from https://doi.org/10.5281/zenodo.5907527. We made available all the data generated in this study at the Zenodo repository https://doi.org/10.5281/zenodo.10710444. This repository includes the synthetic and Cur ground truth networks, the associated simulated scRNA-seq data and pseudo time files, the networks inferred by the four algorithms that we used, and the raw evaluation results (i.e. the values of the topological metrics) for all the networks we analyzed.

## Code availability

The STREAMLINE pipeline in Python, the code for reproducing the figures in R and a tutorial for using the pipeline are available at https://github.com/ScialdoneLab/STREAMLINE. The python pipeline is using Networkx (Hagberg *et al.* 2008) and graph-tool (http://figshare.com/articles/graph_tool/1164194) for graph analysis and handling.
