# Supplementary material for: Topological benchmarking of algorithms to infer gene regulatory networks from single-cell RNA-seq data
Source: Bioinformatics. 2024 Apr 16;40(5):btae267. doi: 10.1093/bioinformatics/btae267 (PMC11096270; doi:10.1093/bioinformatics/btae267)
Supplement: btae267_Supplementary_Data [file btae267_supplementary_data.zip › STREAMLINE_Supplementary_Material.pdf]

Supplementary Material

S1. Parameters of the reference networks

The following two tables summarize the properties of the ground truth and silver standard networks. For the synthetic networks, we list the parameters that were used for sampling as well as the number of networks that resulted. For the experimental networks, the number of cells and genes are shown as well as the number of reference databases that were used. A more elaborated discussion of the properties of the real datasets is given by [McCalla et al., 2023], who collected them.

network type	size	#networks	#nodes	#edges	parameters
Random (ER)	small	20	15	50	none
	large	30	25	100	none
Scale-Free (SF)	small	45	15	50	$\alpha_{in} \in [0.2, 0.5, 0.8]$<br>$\alpha_{out} \in [0.2, 0.5, 0.8]$
	large	90	25	100	$\alpha_{in} \in [0.2, 0.5, 0.8]$<br>$\alpha_{out} \in [0.2, 0.5, 0.8]$
Semi-Scale-Free (SSF)	small	15	15	50	$\alpha_{out} \in [0.2, 0.5, 0.8]$
	large	30	25	100	$\alpha_{out} \in [0.2, 0.5, 0.8]$
Small-World (SW)	small	30	15	45	$\rho \in [0.05, 0.1, 0.2]$
	large	30	25	100	$\rho \in [0.05, 0.1, 0.2]$

Supplementary Table S1: Parameters used to sample synthetic networks. We used all possible combinations which resulted in 290 sampled networks in total.

cell type	#cells	#genes	#reference databases
yeast [Gasch et al., 2017]	163	3847	7
mDC [Shalek et al., 2014]	1211	9411	3
mESC [Tran et al., 2019]	2369	6618	10
hESC [Han et al., 2018]	5520	7468	8

Supplementary Table S2: Properties of the experimental datasets and the number of reference databases used as silver standards.

S2. Binary Edge Detection

The results of our statistical benchmarking, shown in Supplementary Figure S1A, reproduce the previous observation that inference accuracy in terms of AUPRC, AUROC and EPr is moderate at best [Pratapa et al., 2020; Chen et al., 2018]. For synthetic data (Supplementary Figure S1A), the highest prediction accuracy was achieved on Small-World networks, while on Scale-Free, Semi-Scale-Free and Erdos-Renyi networks the performance is worse. Regarding the performance

of the specific algorithms, GRNBoost2, PIDC and PPCOR perform similarly, while SINCERITIES identifies fewer correct interactions. This trend transfers to experimental data (Supplementary Figure S1A). Additionally, the statistical performance on the yeast dataset is worse than on mDC, mESC and hESC. The heatmap in Supplementary Figure S1B-C shows the results of a correlation analysis between the topological metrics employed in our study and the binary edge prediction metrics.

S3. Topological properties of the reference and inferred networks

Figure S2 depicts the topological properties of the sampled ground truth networks as well as the silver standards for the experimental datasets. Among the networks we simulated data from, the Assortativity of SW and ER networks is relatively closer to 0 on average compared to other networks, while the Clustering Coefficient of the ER networks is the lowest. Conversely, SF and SSF have lower (negative) Assortativity and larger Clustering Coefficients together with the SW networks. This is in agreement with the known properties of each class of networks [Ouma et al., 2018]. We report the distribution of the actual values of each metric for each GRN inference algorithm.

S4. Benchmarking results for the Curated networks

In Supplementary Figure S3 we report the ground truth and inferred networks for the Curated dataset Gonadal Sex Development (GSD). The benchmarking results for all the Curated networks are shown in Supplementary Table S3.

Supplementary Table S3: Benchmarking results for the Curated networks for the information exchange, hub topology and binary edge detection metrics.

S5. Benchmarking results for directed networks

In Supplementary Figure S4 we show the distributions of the ground truth values when taking into account edge direction for synthetic and experimental data and the bar plots showing the MSE for the estimations of the Assortativity, Clustering Coefficient and In Centralization, for the GRN inference algorithms that output directed networks (GRNBoost2 and SINCERITIES).

S6. Supplementary results for the Hub Identification metrics

In Supplementary Figure S5 we show the actual values of the Jaccard coefficient for the four Hub Identification metrics that we considered, in synthetic and experimental datasets. In Supplementary Figure S6 we report the results on Hub Identification for directed networks.

S7. Final ranking for directed networks

In Supplementary Figure S7 we show the final ranking of the GRN inference algorithms that output directed networks (GRNBoost2 and SINCERITIES).

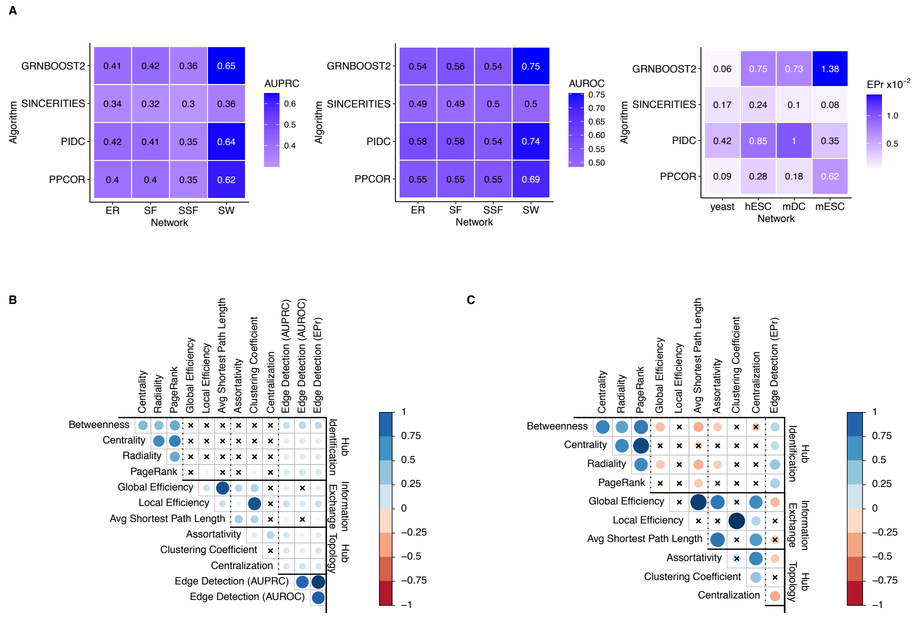

Supplementary Figure S1: A. Benchmarking of edge detection of GRN inference algorithms on synthetic and experimental scRNA-seq datasets. The heatmaps report the median AUPRC and AUROC for synthetic data and the median EPr for experimental data. Rows and columns correspond to GRN inference algorithms and network types, respectively. B-C: Correlation analysis between the topological metrics and the edge detection metrics for the synthetic (panel B) and experimental (panel C) datasets. The heatmaps show Spearman's correlation between the performance scores obtained for each of the indicated metrics (see Methods section).

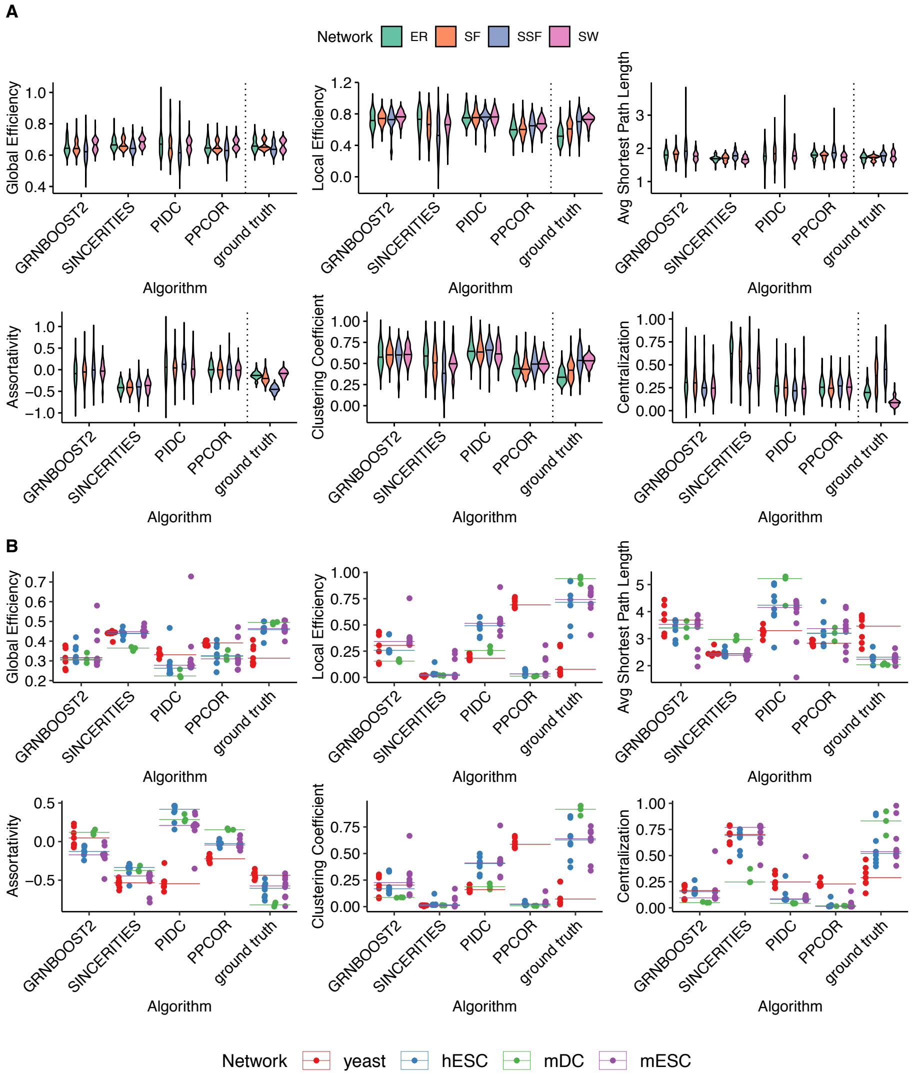

Supplementary Figure S2: Distribution of the topological properties for the synthetic (panel A) and experimental (panel B) datasets. The distributions of the values for the ground truth synthetic networks and silver standard experimental networks are shown on the right of each panel, while the distributions of the inferred values for each GRN inference algorithm are shown on the left. The horizontal lines in panel B represent the median of the metrics for the different types of network.

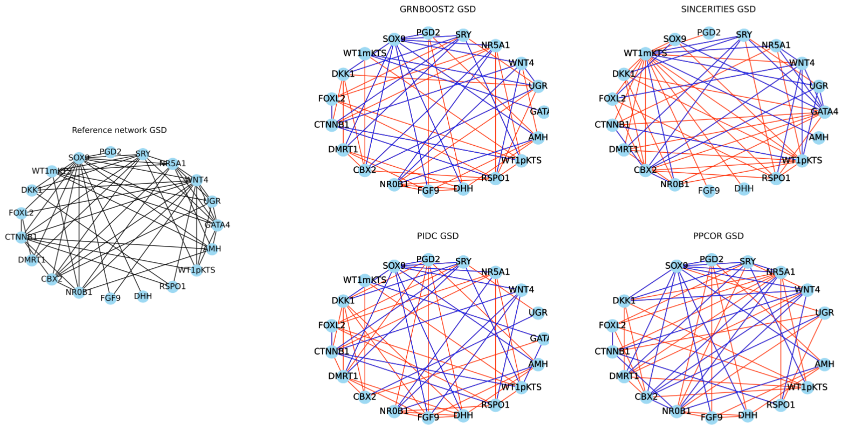

Supplementary Figure S3: Ground truth network for the Gonadal Sex Development (GSD) Curated dataset (left) and corresponding networks inferred by each GRN inference algorithm (right). In the processed inferred networks, the correctly inferred edges are shown in blue and the incorrectly predicted ones are shown in red.

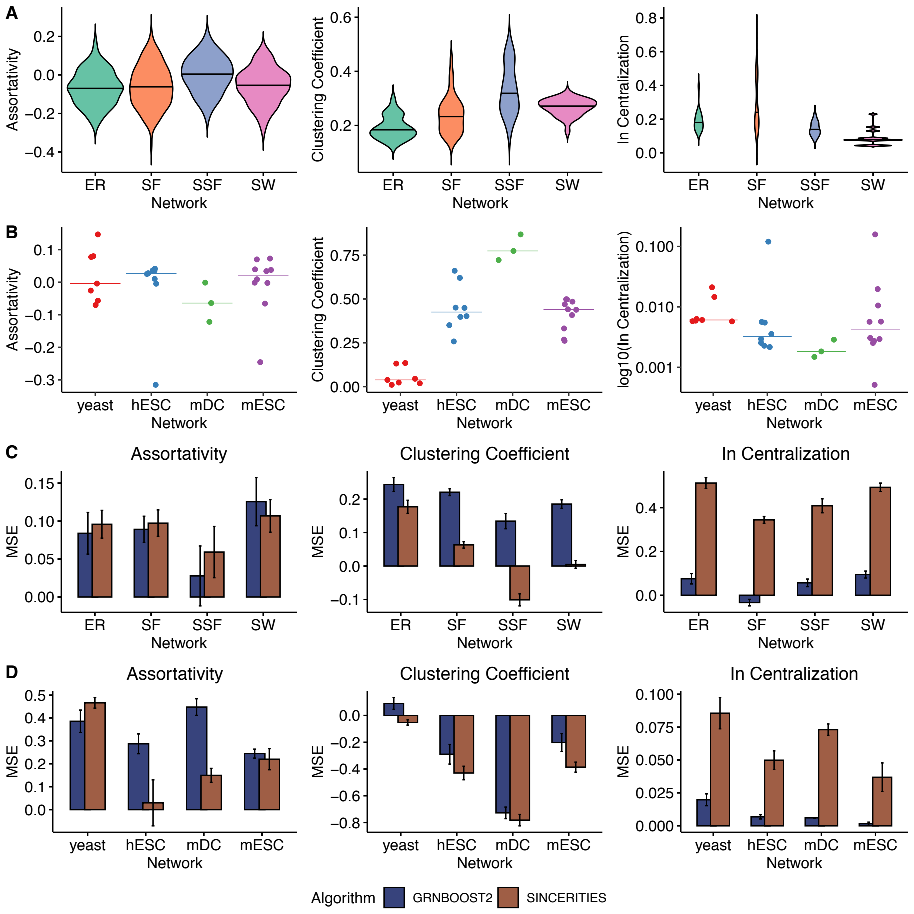

Supplementary Figure S4: A-B. Distributions of the ground truth values of the Assortativity, Clustering Coefficient and In Centralization for synthetic (panel A) and experimental (panel B) datasets, when considering directed ground truth networks. C. Barplots showing the Mean Signed Error (MSE) for the estimations of the topological properties written at the top in different types of synthetic networks (indicated on the x-axis) and for the algorithms that output directed networks (GRNBoost2 and SINCERITIES, marked by colors). D. Same as C, for networks estimated from real scRNA-seq datasets (indicated on the x-axis). The horizontal lines in panel B represent the median of the metrics for the different types of networks. The heights and the error bars display the mean of the MSE values and the standard error of the mean, respectively, computed across datasets and networks in panel C and across networks in panel D.

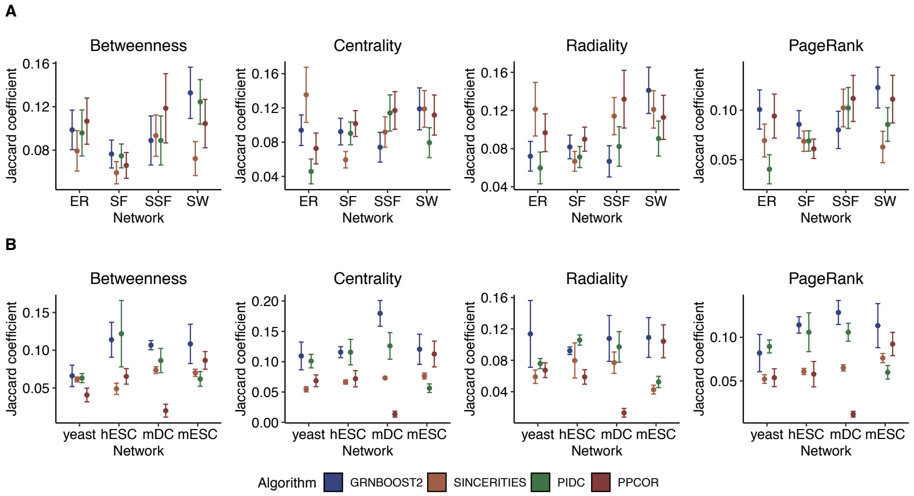

Supplementary Figure S5: Point plots showing the average Jaccard Coefficient for each GRN inference algorithm (marked by colors) obtained using different Hub Identification metrics, for the synthetic (panel A) and experimental (panel B) datasets. The points and the error bars display the mean of the Jaccard coefficient and the standard error of the mean, respectively, computed across datasets and networks in panel A and across networks in panel B.

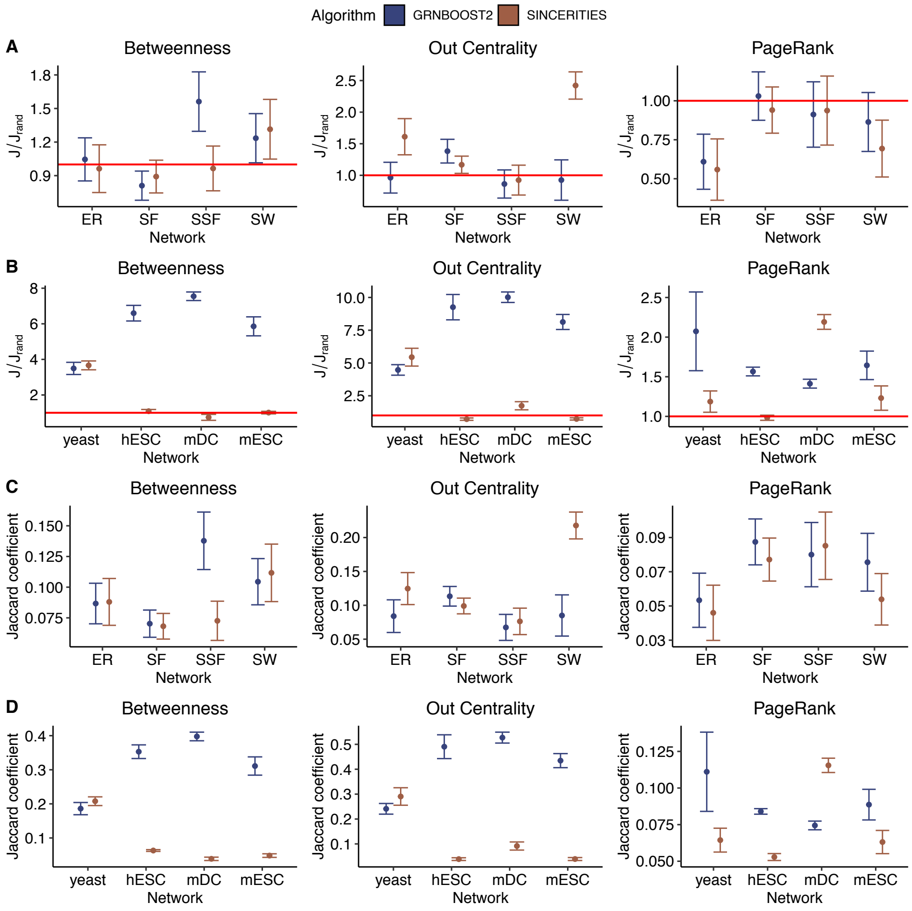

Supplementary Figure S6: A-B: Bar plots showing the average Jaccard Coefficient Ratio for the GRN inference algorithms that output directed networks (GRNBoost2 and SINCERITIES, marked by colors) obtained using different Hub Identification metrics, for the synthetic (panel A) and experimental (panel B) datasets. C-D: Same as A-B for the Jaccard Coefficient. The points and the error bars display the mean of the Jaccard coefficient ratio (A-B) or Jaccard coefficient (C-D) and the standard error of the mean, respectively, computed across datasets and networks (panels A-C) and across networks (panels B-D)).

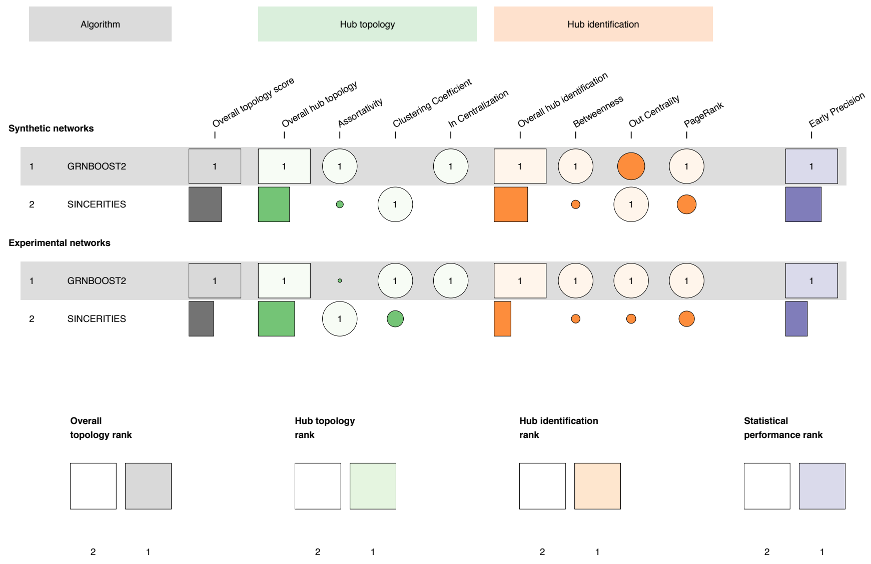

Supplementary Figure S7: Ranking of the GRN inference algorithms for directed ground truth networks. We report the overall performance of the algorithms on each topological metric for synthetic (top rows) and experimental (bottom rows) datasets. The algorithms are ranked according to an overall topology score (see Methods). We also show the ranking for each group of topological metrics (Information Exchange, Hub Topology and Hub Identification) and we report the performance in binary edge detection in the last column. The legend at the bottom shows the association between the colors and the ranks for each group of metrics.
